# *CFH* Y402H Confers Similar Risk of Soft Drusen and Both Forms of Advanced AMD

**DOI:** 10.1371/journal.pmed.0030005

**Published:** 2005-11-29

**Authors:** Kristinn P Magnusson, Shan Duan, Haraldur Sigurdsson, Hjorvar Petursson, Zhenglin Yang, Yu Zhao, Paul S Bernstein, Jian Ge, Fridbert Jonasson, Einar Stefansson, Gudleif Helgadottir, Norman A Zabriskie, Thorlakur Jonsson, Asgeir Björnsson, Theodora Thorlacius, Palmi V Jonsson, Gudmar Thorleifsson, Augustine Kong, Hreinn Stefansson, Kang Zhang, Kari Stefansson, Jeffrey R Gulcher

**Affiliations:** 1DeCODE Genetics, Reykjavik, Iceland; **2**Department of Ophthalmology and Visual Science, Moran Eye Center, University of Utah, Salt Lake City, Utah, United States of America; **3** Program in Human Molecular Biology and Genetics, Eccles Institute of Human Genetics, University of Utah, Salt Lake City, Utah, United States of America; **4**Zhongshan Ophthalmic Center, Sun Yat-Sen University, Gaung Zhou, China; **5**Department of Ophthalmology, National University Hospital, Reykjavik, Iceland; **6**Faculty of Medicine, University of Iceland, Reykjavik, Iceland; **7**Sichuan Academy of Medical Sciences and Sichuan Provincial People's Hospital, Chengdu, China; **8**Department of Geriatrics, National University Hospital, Reykjavik, Iceland; Southampton General HospitalUnited Kingdom

## Abstract

**Background:**

Age-related macular degeneration (AMD) is the most common cause of irreversible visual impairment in the developed world. The two forms of advanced AMD, geographic atrophy and neovascular AMD, represent different pathological processes in the macula that lead to loss of central vision. Soft drusen, characterized by deposits in the macula without visual loss, are considered to be a precursor of advanced AMD. Recently, it has been proposed that a common missense variant, Y402H, in the Complement Factor H *(CFH)* gene increases the risk for advanced AMD. However, its impact on soft drusen, GA, or neovascular AMD—or the relationship between them—is unclear.

**Methods and Findings:**

We genotyped 581 Icelandic patients with advanced AMD (278 neovascular AMD, 203 GA, and 100 with mixed neovascular AMD/GA), and 435 with early AMD (of whom 220 had soft drusen). A second cohort of 431 US patients from Utah, 322 with advanced AMD (244 neovascular AMD and 78 GA) and 109 early-AMD cases with soft drusen, were analyzed. We confirmed that the *CFH* Y402H variant shows significant association to advanced AMD, with odds ratio of 2.39 in Icelandic patients (*p* = 5.9 × 10^−12^) and odds ratio of 2.14 in US patients from Utah (*p* = 2.0 × 10^−9^) with advanced AMD. Furthermore, we show that the Y402H variant confers similar risk of soft drusen and both forms of advanced AMD (GA or neovascular AMD).

**Conclusion:**

Soft drusen occur prior to progression to advanced AMD and represent a histological feature shared by neovascular AMD and GA. Our results suggest that *CFH* is a major risk factor of soft drusen, and additional genetic factors and/or environmental factors may be required for progression to advanced AMD.

## Abstract 2

A common missense variant, Y402H, in the Complement Factor H gene is associated strongly with soft drusen, a precursor of advanced age-related macular degeneration

## Introduction

Age-related macular degeneration (AMD) includes a wide range of phenotypes. Early AMD is characterized mainly by the presence of soft drusen in the macula without visual loss, while advanced AMD is characterized by geographic atrophy (GA or dry AMD) and neovascular AMD (wet AMD) with visual loss. Despite the rising prevalence of AMD as a result of increasing life expectancy, its underlying pathogenesis is poorly understood and there are limited treatment options available. Nutritional supplements and antagonists of vascular endothelial growth factor have been reported to decrease visual loss in neovascular AMD somewhat [1]. Drusen are comprised of small yellowish, extracellular deposits of lipid, protein, and cellular debris, formed beneath the retinal pigment epithelium (RPE), a tissue that underlies the photoreceptor cells. Biochemical analysis of drusen have indeed resulted in identification of complement components and inflammatory modulators [2–8]. Soft drusen and pigmentary abnormalities of the RPE are considered to be an early indication of risk of developing advanced AMD. GA is a consequence of the degeneration of the photoreceptor cells and the RPE. Neovascular AMD is characterized by abnormal growth of capillaries from the choroid and by subsequent exudation of fluid, lipid, and blood.

Several genome-wide linkage scans for AMD including ours (unpublished data) have found suggestive linkage on chromosome 1q [9–15]. The addition of markers within the linkage peak led to recent reports by five groups that there is an association between a common missense variant (Y402H) in *CFH* and AMD in the United States [3,16–19]. The Y402H allele was present in at least 60% of AMD patients with risk ratios of between 2.0 and 3.5 for one risk allele, and with risk ratios of between 3.3 and 7.4 for carriers of two risk alleles. It has been postulated that the Y402H variant may lead to decreased binding to CRP and heparin and therefore less inhibition of the complement pathway, causing overactivity and deposition of the complement pathway proteins [20]. CFH protein has been detected in choriocapillaris and within soft drusen [3].

However, the question of how the Y402H allele contributes to the different subtypes of AMD has not been properly addressed, owing either to insufficient clinical information or to sample size, as neovascular AMD dominates the patient cohorts in most previously reported studies [3,16–19].

In order to investigate the association between *CFH* and AMD we performed genotype–phenotype correlations on different clinical subtypes of early and advanced AMD in US and European populations.

## Methods

### Patients

This study was approved by the Data Protection Authority of Iceland and the National Bioethics Committee of Iceland, and the Institutional Review Board of the University of Utah. All participants signed written informed consent prior to participation in the study. All personal identifiers associated with blood samples, medical information, and genealogy were encrypted. For samples from Iceland, encryption was carried out by the Data Protection Authority, using a third-party encryption system [21]. The Icelandic cohort recruited by DeCODE Genetics has detailed phenotypic information for 2,220 individuals, ie 1,112 patients with neovascular AMD, GA, or early AMD, and 1,108 of their unaffected relatives. Probands were recruited from a list of 2,840 consecutive patients diagnosed with AMD or early AMD at the University Eye Clinic, Reykjavik, or listed in the Icelandic Registry for the Blind during the years 1980–2001, together with relatives. Population controls were not related to the AMD cohort and did not include any first- or second-degree relative pair. A second control group (longevous controls) included 171 unrelated individuals, aged 90 y or older, who had no signs of advanced AMD, diagnosed based on their ability to see fine detail, including print, as assessed in Section D1 of the Minimum Data Set of the Resident Assessment Instrument [22]. Since the prevalence of AMD increases dramatically with age, this group represents healthy “supercontrols” for AMD. A second sample of AMD patients were recruited in Utah at the Moran Eye Center of the University of Utah, and were age-matched with controls with normal eye examinations (individuals aged 60 y or older, with no drusen or RPE changes).

All participants in both populations went through a standard examination protocol and visual-acuity measurements. Slitlamp biomicroscopy of the fundi using 90-diopter lenses was performed. A pair of stereoscopic color fundus photographs (50°) were taken, centered on the fovea using a Topcon fundus camera (Topcon TRV-50VT, Topcon Optical Company, Tokyo, Japan) by a trained ophthalmic photographer. Grading was carried out using a standard grid classification suggested by the International Age-Related Maculopathy Epidemiological Study Group for age-related maculopathy and AMD [23]. All abnormalities in the macula were recorded, including type, size, and number of drusen as well as the presence of hyperpigmentation and hypopigmentation, together with advanced AMD.

### Genotyping

The Icelandic cohort that was genotyped included 581 patients with advanced AMD and 435 patients with early AMD, and allele frequencies were compared to that of either 891 population controls or 171 longevous healthy controls (Table 1). The Utah cohort of 244 patients with neovascular AMD, 78 patients with GA, and 109 patients with early AMD with soft drusen was genotyped, and allele frequencies were compared to 203 age-matched healthy controls. A TaqMan assay (Applied Biosystems, Foster City, California, United States) was performed on a 384-well GeneAmp PCR System 9700 (Applied Biosystems) used for PCR to genotype the Icelandic cohort. A direct DNA- sequencing method was used on an ABI 3100 genetic analyzer (Applied Biosystems) to genotype the Utah cohort.

**Table 1:**
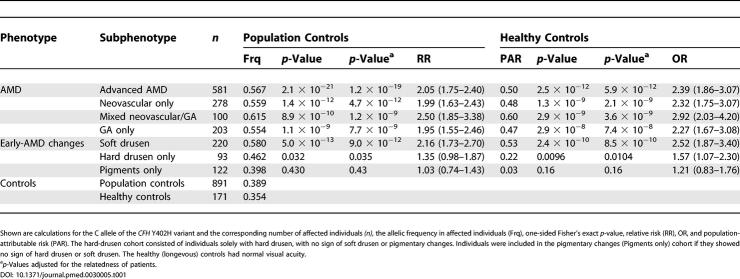
Association between Subphenotypes of AMD and *CFH* Y402H Variant in the Icelandic Cohort

### Data Analysis

For the single-marker association of the *CFH* Y402H variant (rs1061170), we used Fisher's exact test to calculate one-sided *p*-values for the at-risk allele. As the patient cohort was recruited as families for a linkage analysis, we also repeated the test for association, correcting for the relatedness of the patients by extending a variance-adjustment procedure described previously [24] for sib-ships to apply to general familial relationships. Using the variance-adjustment procedure, the variance of the test statistic is adjusted to take into account the decrease in the effective sample size resulting from the fact that genotypes of relatives are not independent. Both unadjusted and adjusted *p*-values are presented for comparison. We calculate the odds ratio (OR) of the frequency of the at-risk allele as OR = *p*/(1−*p*)/*s*/(1−*s*), where *p* and *s* are the frequency of the at-risk allele in the patients and in the controls, respectively. In the case of population controls and assuming the multiplicative model, in which the risks of the two alleles of the single-nucleotide polymorphism a person carries multiply [25,26]*,* this corresponds to an estimate of the relative risk of the mutation compared to the wild-type. Specifically, with population controls and the multiplicative model, it can be shown through Bayes' Rule that the OR, as defined above, corresponds to Risk(CT)/Risk(TT) = Risk(CC)/Risk(CT), where C is the mutated allele and T the wild-type, and Risk is the probability of disease given the genotype.

On the basis of the frequency of the at-risk allele and the relative risk, we calculate the population-attributable risk or the reduction in the number of disease cases if the at-risk allele was removed from the population, again assuming the multiplicative model. Confidence intervals of relative risks and ORs were based on the variance-adjusted tests for association, assuming a log-normal distribution. To avoid confusion and to be consistent, we report the results as OR when using healthy controls and as relative risk when using population controls.

## Results

In agreement with previous reports [3–19], the Y402H allele confers an OR of 2.32 when comparing Icelandic patients with neovascular AMD to healthy controls. Based on the comparison with population controls, the relative risk of the mutation is estimated to be 1.99 with a corresponding estimated population-attributable risk of 0.48. The Y402H variant also contributes to GA, with OR of 2.27. The patient group with mixed GA/neovascular AMD gave similar results with OR of 2.92. Thus, the Y402H allele contributes equally to GA and neovascular AMD in Icelandic patients with advanced AMD (Table 1).

The comparable association to neovascular AMD and GA was replicated in the Utah cohort, giving ORs of 2.17 and 2.05, respectively (Table 2). Therefore, we conclude that the *CFH* variant contributes equally to GA and neovascular AMD in our European and US cohorts.

**Table 2:**
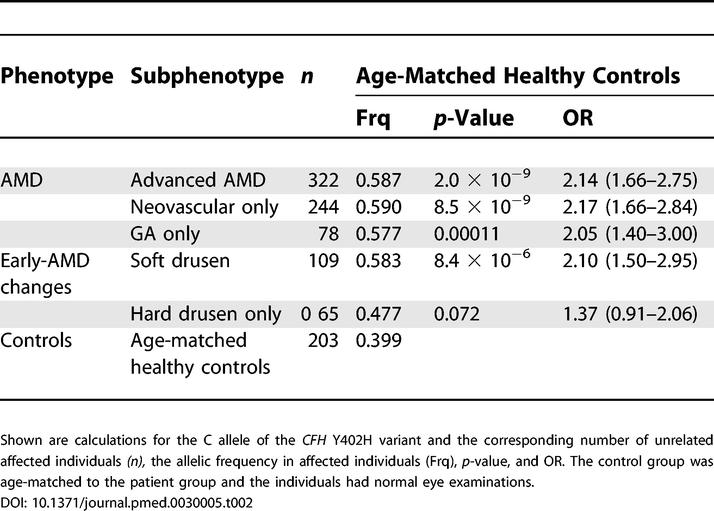
Association between Subphenotypes of AMD and *CFH* Y402H Variant in the Utah Cohort

Furthermore, the Y402H variant contributes to soft drusen in early AMD, with similar ORs in the Icelandic and Utah study groups of 2.52 and 2.10, respectively (see Tables 1 and 2). In contrast, the variant does not show significant association to pigmentary changes found in early AMD. In Iceland we observed significant association (*p* = 0.01) to hard drusen but with a lower OR (1.57) (Table 1). A significant difference in *CFH* Y402H allele frequencies was observed when patients with soft drusen were compared with an unrelated set of patients with hard drusen (*p* = 0.011). This *CFH* variant also confers increased risk although this is not significant when comparing hard drusen to controls in the Utah cohort (see Table 2).

We also typed the *CFH* Y402H allele in four ethnically diverse populations from the International HapMap project [27]; Caucasians, residents of Utah with ancestry from northern and western Europe (59), Yorubians, residents of Nigeria (57), Japanese (31), and Chinese (44). The allele frequencies for *CFH* Y402H in Caucasians and Africans were similar, 39% versus 30.7%, while they were much lower in Asians—8.1% in the Japanese and 6.8% in the Chinese.

## Discussion

We have shown that the *CFH* Y402H allele confers significant risk to neovascular AMD, GA, and soft drusen in early AMD in US and European Caucasian populations. The effect of the *CFH* Y402H allele on soft drusen in early AMD is similar to its effect on the advanced forms of AMD, neovascular AMD, and GA. Advanced AMD is considered to be one disease with two different end-stage lesions, i.e., choroidal neovasculariztion and GA. Leaky choroidal neovascular blood vessels between Bruch's membrane and RPE are seen typically in neovascular AMD, while GA is characterized by RPE atrophy and overlying photoreceptor loss. Soft drusen located between the RPE and Bruch's membrane are usually precursors of both forms of advanced AMD. The 5-y incidence of soft drusen in the Reykjavik Eye Study, a population-based epidemiological study of individuals aged 50 y and older [28,29], was found to be similar to that of the Beaver Dam Eye Study in the US [30], and the Blue Mountains Eye Study in Australia [31]. Neovascular AMD outnumbers GA by approximately three to one in both the Beaver Dam Eye Study and the Blue Mountains Eye Study. Similar figures were seen in most other Caucasian populations. In the Icelandic population under consideration here, however, GA was found to outnumber neovascular AMD by three to one, as reported in the Reykjavik Eye Study [28,29].It is an open question whether the high GA/neovascular AMD ratio in Iceland is due to genetic or environmental factors that increase the risk of GA or decrease the risk of neovascular AMD. Consumption of fresh fish and fishliver oil with omega-3 polyunsaturated fatty acids among Icelanders is among the highest in the world [28]. Interestingly, Seddon et al. [32] reported a trend for decreasing odds of neovascular AMD with increasing amounts of omega-3 and fresh-fish intake.

The discovery of *CFH* as an important AMD gene, contributing to the common form of AMD and its confirmation by several groups, is a major advance towards understanding the genetic risk and pathogenesis of AMD. It is apparent that this single common variant confers similar risk in all of the US populations tested and, furthermore, our result for the association of the *CFH* variant to advanced AMD in a European population is comparable to that in the US. However, the previously reported studies had not adequately addressed the effect of the *CFH* variant on GA or early AMD. We therefore tested the reported variant in the *CFH* region for association to the subtypes of AMD in both of our cohorts.

Functionally, *CFH* is thought to aid in keeping the complement pathway of the innate immune system in check. It is tempting to postulate that a hypothetical lower activity of complement H protein with the histidine variant may lead to increased inflammation that would contribute to the neovascular form of AMD as suggested before [17]. Alternatively, others have suggested that it may have a direct role in soft-drusen formation, which may also be linked to inflammation [3]. Our results, showing that the *CFH* variant contributes equally to GA and to neovascular AMD, would tend to refute the first hypothesis and lend support to the second.

Given that the protein component of soft drusen includes members of the complement system (including Complement H), we tested the effect of the *CFH* variant on risk of soft drusen without advanced AMD. Interestingly, this *CFH* variant confers risk of soft drusen with similar OR (2.52) as with both forms of advanced AMD, even before the fundoscopic findings and visual loss fulfil the criteria for advanced AMD in two independent cohorts. Conversely, there is little or no impact of the *CFH* variant on other features such as pigmentary changes and hard drusen. Therefore, it appears that the Y402H variant in *CFH* contributes to the increased risk of advanced AMD largely or entirely through its impact on the development of soft drusen as a precursor of advanced AMD.

This observation may not be surprising given that soft drusen is comprised, in part, of complement proteins including *CFH* and its binding partner, complement 3b [20]. However, many more elderly patients develop soft drusen than those who ultimately progress to advanced AMD. Numerous epidemiological studies have shown that the prevalence of soft drusen is two to three times greater than that of advanced AMD [33]. The prevalence of soft drusen in Caucasian populations increases with age at the same rate as AMD. Most patients with soft drusen are without any visual symptoms for decades, and only a fraction of individuals who have soft drusen will eventually progress to AMD with visual-acuity loss. For example in the Beaver Dam eye study, only 14% of the patients with soft drusen developed AMD over a 10-y period [30]. Interestingly, in persons of African origin living in United States and Barbados, prevalence of early AMD and soft drusen is slightly lower than in whites, but advanced AMD is rare [34,35]. The allele frequency of the Y402H is less (0.31 versus 0.39) in African Americans than in Caucasians. Therefore, the prevalence discrepancy between the early and advanced AMD in African Americans is consistent with our hypothesis that *CFH* Y402H causes soft-drusen formation, but it is not sufficient for progression to advanced AMD. To substantiate this hypothesis further, it will be interesting to correlate the prevalence of soft drusen with advanced AMD in Asian populations. Our analysis of the *CFH* Y402H variant demonstrated that its frequency is less in Asian populations: 0.08 in the Japanese samples and 0.07 in the Chinese. Indeed, AMD in Asians is considered to be infrequent, but careful genotype–phenotype correlation studies are needed in non-Caucasian populations.

Significant genetic influence in early AMD has been demonstrated in a classical twin study comparing concordance of 226 monozygotic and 280 dizygotic twin pairs, with soft drusen and multiple hard drusen showing strong genetic influences with heritability of 57% and 81%, respectively [36]. This is comparable to the heritability of advanced AMD [37]. However, we show that the *CFH* variant is a risk factor for soft drusen, but not for hard drusen or pigmentary changes, per se. Therefore, there may be other genes that influence the appearance of hard drusen and pigmentary changes. In addition, the difficulty in explaining the difference in the ratio of the prevalences in neovascular AMD and GA across populations through the *CFH* variant alone supports the notion that additional genetic or environmental factors contribute to the pathogenesis. It is likely that there are other important genes, yet to be found, that contribute to the risk of advanced AMD, particularly among those who already have soft drusen.

## Supporting Information

### Accession Numbers

The LocusLink (http://www.ncbi.nlm.nih.gov/entrez) accession number for the gene discussed in this paper, *CFH,* is ID 3075. The OMIM (http://www.ncbi.nlm.nih.gov/entrez) identification number for AMD, ARMD1, is 603075; and for the *CFH* gene is 134370.

**Patient Summary**

**Background**

The commonest cause of poor eyesight in later life in the developed world is known as age-related macular degeneration (AMD). The macula is the central part of the retina (the film-like membrane at the back of the eye) which is the most sensitive and important for sharp central vision. An early sign of AMD is what are called “drusen”—deposits of protein, fat, and cells—which doctors can see in the back of the eye. There are two types of advanced AMD: so-called “wet” or neovascular AMD (neovascular means “new vessel”) and “dry” or geographic atrophy AMD (atrophy means to waste away). Wet AMD occurs when abnormal, fragile blood vessels grow under the macula behind the retina. These blood vessels often leak blood and fluid, which lift the macula. Dry AMD occurs as the light-sensitive cells in the macula (the rods and cones) break down.

**Why Was This Study Done?**

Although this disease is common, little is understood about why it occurs, and current treatments have limited efficacy. Previous studies have suggested that a gene in a particular part of Chromosome 1 is linked to the chance of getting AMD. The responsible gene is Complement Factor H *(CFH),* which codes for a protein that is involved in keeping one part of the immune system in check. A variant of *CFH* has been previously shown to be present more frequently in people with advanced AMD compared to normal controls. These investigators wanted to go further, to find out whether this variant was more linked to the wet or to the dry type of AMD and to early AMD.

**What Did the Researchers Do and Find?**

They looked at the variant of *CFH* in two groups of patients with various types of AMD, 1,118 from Iceland and 431 from Utah, and compared the results with people without AMD from the same ethnic groups and age. As had been shown before, they found that one variant of this gene occurred more frequently in the wet form of AMD. However, they report two new observations. First, the variant of *CFH* also confers risk for the dry form of AMD and second, the variant confers similar risk to drusen in the early form of AMD.

**What Do These Findings Mean?**

It appears that this gene variant is important early on in the development of AMD—which makes sense as the protein for which this gene codes is involved in keeping the immune system under control. The particular variant found here may not be as efficient as the normal one—that is, it makes it more likely that inflammation will develop in the eye. These findings do not have any immediate implications for treatment, but they suggest that there are other genes that cause the severe forms of AMD with blindness.

**Where Can I Get More Information Online?**

Here are several Web sites with information on macular degeneration.

MedlinePlus:

http://www.nlm.nih.gov/medlineplus/ency/article/001000.htm

National Institutes of Health Senior Health:

http://nihseniorhealth.gov/agerelatedmaculardegeneration/toc.html

National Eye Institute:

http://www.nei.nih.gov/health/maculardegen/armd_facts.asp

Prevent Blindness America:

http://www.preventblindness.org/eye_problems/amdFAQ.html

Foundation Fighting Blindness:

http://www.blindness.org/MacularDegeneration/

## Abbreviations

AMD: age-related macular degeneration
GA: geographic atrophy
OR: odds ratio
RPE: retinal pigment epithelium
